# Early decrease in erector spinae muscle area and future risk of mortality in idiopathic pulmonary fibrosis

**DOI:** 10.1038/s41598-020-59100-5

**Published:** 2020-02-11

**Authors:** Akiko Nakano, Hirotsugu Ohkubo, Hiroyuki Taniguchi, Yasuhiro Kondoh, Toshiaki Matsuda, Mitsuaki Yagi, Taiki Furukawa, Yoshihiro Kanemitsu, Akio Niimi

**Affiliations:** 1 Department of Respiratory Medicine, Allergy and Clinical Immunology, Nagoya City University Graduate School of Medical Sciences, Nagoya, Japan; 2 Department of Respiratory Medicine and Allergy, Tosei General Hospital, Nagoya, Japan; 3 Department of Respiratory Medicine, National Hospital Organization Higashinagoya National Hospital, Nagoya, Japan; 4 Department of Respiratory Medicine, Nagoya University Graduate School of Medicine, Nagoya, Japan

**Keywords:** Respiratory tract diseases, Medical research

## Abstract

Computed tomography (CT) assessment of the cross-sectional area of the erector spinae muscles (ESM_CSA_) can be used to evaluate sarcopenia and cachexia in patients with lung diseases. This study aimed to confirm whether serial changes in ESM_CSA_ are associated with survival in patients with idiopathic pulmonary fibrosis **(**IPF). Data from consecutive patients with IPF who were referred to a single centre were retrospectively reviewed. We measured the ESM_CSA_ at the level of the 12th thoracic vertebra on CT images at referral and 6 months later (n = 119). The follow-up time was from 817–1633 days (median, 1335 days) and 59 patients (49.6%) died. A univariate Cox regression analysis showed that the decline in % predicted forced vital capacity (FVC) (Hazard ratios [HR] 1.041, 95% confidence interval [CI] 1.013–1.069, P = 0.004), the decline in body mass index (BMI) (HR 1.084, 95% CI 1.037–1.128; P < 0.001) and that in ESM_CSA_ (HR 1.057, 95% CI 1.027–1.086; P < 0.001) were prognostic factors. For multivariate analyses, the decline in ESM_CSA_ (HR 1.039, 95% CI 1.007–1.071, P = 0.015) was a significant prognostic factor, while those in % FVC and BMI were discarded. Early decrease in ESM_CSA_ may be a useful predictor of prognosis in patients with IPF.

## Introduction

Idiopathic pulmonary fibrosis (IPF) is a fibrotic pulmonary disease which leads to the death of most patients^1,2^. The prognosis for IPF patients is poor at 3 to 4 years^1–4^. The disease is characterised by decreased lung volumes and reduced gas exchange, and it is associated with symptoms of progressive dyspnoea, cough and reduced exercise capacity. Several prognostic factors for IPF are known, including dyspnoea score, pulmonary function, oxygen desaturation during exercise, and fibrotic changes on high resolution computed tomography (HRCT)^5^. Other prognostic factors have been reported, such as pathological findings^6,7^, serum biomarkers^8,9^, St. George’s Respiratory Questionnaire score^10^, fibrosis score^11^, normal lung volume on HRCT^12,13^ and mean pulmonary arterial pressure^14^. Among pulmonary function variables, a decline in forced vital capacity (FVC) is widely known as a prognostic factor^15,16^.

Cachexia is a complex metabolic syndrome. It is associated with underlying diseases and is characterised by loss of muscle with or without loss of fat mass in cancer and chronic diseases such as congestive heart failure, chronic kidney disease and chronic obstructive pulmonary disease (COPD)^17,18^. Loss of skeletal muscle in cancer patients can potentially be due to anorexia and early satiety, reduced muscle protein synthesis, and/or increased muscle protein breakdown^19^. Sarcopenia is a syndrome characterised by a progressive and generalised loss of skeletal muscle mass and strength, and it carries a risk of poor outcomes such as physical disability, poor quality of life and death^20^.

Assessment of the cross-sectional area of the erector spinae muscles (ESM_CSA_) from chest computed tomography (CT) scans has been used to evaluate sarcopenia and cachexia in patients with chronic lung disease^21,22^. Compared with healthy individuals, ESM_CSA_ is decreased in patients with IPF, idiopathic pleuroparenchymal fibroelastosis (iPPEE) and chronic obstructive pulmonary disease (COPD)^21,22^. ESM_CSA_ assessed by chest CT is an independent prognostic factor for patients with COPD^21^. Miller *et al*. reported that height-adjusted ESM_CSA_ was significantly associated with 30-day mortality and length of hospital stay after lobectomy in patients with lung cancer^23^. Suzuki *et al*. also revealed that a smaller ESM_CSA_ in patients with IPF was associated with a poor prognosis^22^.

We queried whether the survival of patients with IPF decreases with a decrease in muscle mass. Resultantly, we hypothesised that a relative decline in ESM_CSA_ over the course of 6 months is associated with decreased survival in patients with IPF. We measured the ESM_CSA_ at the level of the spinous process of 12th thoracic vertebra on CT images at baseline (n = 144) and 6 months later (n = 119) in patients with IPF.

## Results

### Patient characteristics and ESM_CSA_

The clinical characteristics and ESM_CSA_ of 119 patients who underwent HRCT after 6 months are shown in Table 1. The baseline ESM_CSA_ was 34.2 [27.7–40.0] cm^2^ whereas ESM_CSA_ after 6 months was 31.6 [25.0–37.0] cm^2^.

**Table 1:** Characteristics and ESM_CSA_ of patients available for CT after 6 months.

Variable	0 month	After 6 months
Total, n	119	119
Age, years	67.0 [61.0–71.0]	
Sex, Female, n (%)	21 (17.6%)	
Never smoker, n (%)	25 (21.0%)	
Ex-smoker, n (%)	81 (68.1%)	
Current smoker, n (%)	13 (10.9%)	
Smoking history, pack-years	36.0 [5.0–55.5]	
Body mass index, kg/m^2^	23.4 [21.7–25.2]	23.1 [21.4–25.4]
Biopsy-proven IPF, n (%)	62 (52.1%)	
FVC, % predicted	84.2 [70.4–96.5]	78.1 [67.5–96.3]
FEV_1_/FVC, %	85.8 [81.3–90.6]	86.2 [79.7–90.7]
DL_CO_, % predicted*	60.7 [48.8–76.7]	58.6 [45.3–69.6]
Distance walked during 6MWT, m	589 [524–645]	
Lowest SpO_2_ during 6MWT, %	85.0 [80.0–89.0]	
ESM_CSA,_ cm^2^	34.2 [27.7–40.0]	31.6 [25.0–37.0]

Data are presented as median [interquartile range] or n (%). Abbreviations: FVC, forced vital capacity; FEV1, forced expiratory volume in 1.0 second; DLCO, diffuse capacity of the lung for carbon monoxide; 6MWT, 6-minute walk test; SpO2, percutaneous oxygen saturation; ESMCSA, cross-sectional area of elector spine muscles. *We analysed using n = 114, because 5 cases were missing.

The correlation of ESM_CSA_ values in the 119 patients between trained individuals were as follows; r = 0.951(95% confidence intervals [CI] 0.913–0.965), P < 0.001. The Bland-Altman analysis revealed that the agreement between two individuals was excellent (0.934).

The clinical characteristics and ESM_CSA_ of 144 patients at baseline, 119 patients who underwent HRCT after 6 months, and 25 patients who did not undergo HRCT after 6 months are shown in Supplemental Table 1. We could not analyse CT in 25 patients after 6 months. Eleven of the 25 patients died; 5 were transferred; 3 were unchanged in pulmonary function test; 2 were out of timing; 1 was rejected; and 1 experienced acute exacerbation. There were significant differences in age (P = 0.001), body mass index (BMI) (P = 0.039), %FVC (P < 0.001), % deffuse capacity of the lung for carbon monoxide (DL_CO_) (P = 0.008), distance walked during the 6 minute walk test (6MWT) (P < 0.001), lowest SpO_2_ during the 6MWT (P = 0.046) and ESM_CSA_ (P = 0.009) between patients who underwent HRCT after 6 months and those who did not.

### The distributions of ESM_CSA_ declines

**Figure 1:**
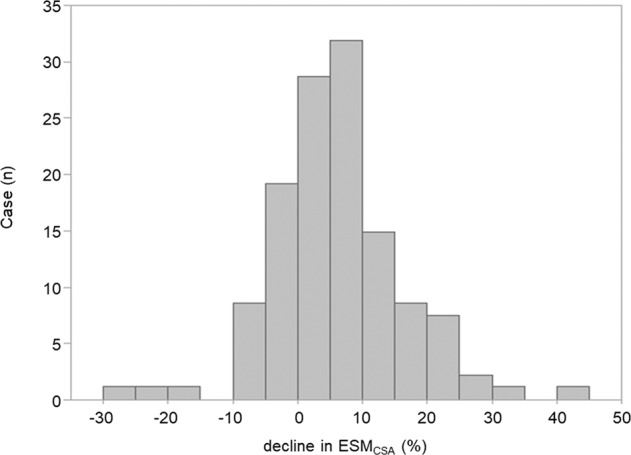
The distribution histogram of the decline in ESM_CSA_. The horizontal axis represents the relative decline in ESM_CSA_ and the vertical axis represents the number of patients.

The distribution of the relative decline in ESM_CSA_ is shown in Fig. 1. We compared the relative decline in %FVC with that in ESM_CSA_ to determine which was greater. We observed that the relative decline in ESM_CSA_ (6.2 [0.5–11.8] %) was significantly greater than that in %FVC (1.4 [−3.1%–5.0] %) (Student’s t-test, P < 0.001).

### Correlations between the decline in ESM_CSA_ and other clinical parameters

**Figure 2:**
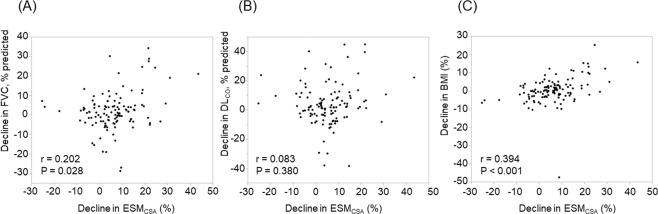
The correlations of decline in ESM_CSA_ with declines in FVC, DL_CO_ and BMI. The correlations of relative decline in ESM_CSA_ with relative declines in FVC (**A**), DL_CO_(**B**) and BMI(**C**) are shown.

The correlations between the relative decline in ESM_CSA_ and other clinical parameters were as follows (Table 2); baseline %FVC (r = −0.285 [−0.464–−0.089], P = 0.002), lowest SpO_2_ during 6MWT (r = −0.290 [−0.458– −0.108], P = 0.002), the relative decline in %FVC (r = 0.202 [0.022–0.358], P = 0.028), and the relative decline in BMI (r = 0.394 [0.217–0.552], P < 0.001). Figure 2 shows the correlations of relative decline in ESM_CSA_ with the relative decline in %FVC, the relative decline in %DL_CO_, and the relative decline in BMI.

**Table 2:** Correlations between the decline in the ESM_CSAs_ and clinical parameters.

Variables	*r*	95%CI	P-value
Age, year	−0.044	−0.244–0.156	0.637
Baseline body mass index, kg/m^2^	−0.175	−0.357–0.020	0.057
Baseline FVC, % predicted	−0.285	−0.464–−0.089	0.002
Baseline FEV_1_/FVC, %	0.187	−0.009–0.369	0.042
Baseline DL_CO_, % predicted	−0.172	−0.337–0.017	0.063
Baseline distance walked during 6MWT, m	−0.171	−0.341–0.019	0.064
Baseline lowest SpO_2_ during 6MWT, %	−0.290	−0.458–−0.101	0.002
Relative decline in FVC, %	0.202	0.022–0.358	0.028
Relative decline in DL_CO_, %*	0.083	−0.096–0.267	0.377
Relative decline in body mass index, %	0.394	0.217–0.552	<0.001

Abbreviations: ESMCSA;, cross-sectional areas of elector spine muscles; FVC, forced vital capacity; FEV1, forced expiratory volume in 1.0 second; DLCO, diffuse capacity of the lung for carbon monoxide; 6MWT, 6-minute walk test; SpO2, percutaneous oxygen saturation.

*We analysed using n = 114 because 5 cases were missing.

The ESM_CSA_ at baseline in 144 patients was 33.3 [25.9–39.1] cm^2^. The correlations between the ESM_CSA_ at baseline and other clinical parameters are shown in Supplemental Table 2.

### Prognostic survey

Fifty-nine of 119 patients (49.6%) died during the study period. The follow-up time of 119 patients was 1335 [817–1633] days. Four cases were lost to follow-up.

### Uni- and multivariate Cox regression analyses

Hazard ratios (HRs) and 95% CIs in the cohort that was available for 6 months of follow-up (n = 119) on univariate and multivariate Cox regression analyses are shown in Table 3. The univariate Cox regression analysis showed that the relative decline in BMI (HR 1.084, 95% CI 1.037–1.128, P < 0.001), the relative decline in % FVC (HR 1.041, 95% CI 1.013–1.069, P = 0.004), and the relative decline in ESM_CSA_ (HR 1.057, 95% CI 1.027–1.086, P < 0.001) were prognostic factors. On multivariate analyses, the relative decline in ESM_CSA_ (HR 1.039, 95% CI 1.007–1.071, P = 0.015) was a significant prognostic factor, while the relative decline BMI (HR 1.036, 95% CI 0.986–1.088, P = 0.163) and the relative decline in %FVC (HR 1.021, 95% CI 0.992–1.050, P = 0.155) were not.

**Table 3:** Prediction of mortality by uni- and multivariate Cox-proportion analyses in the patients available for CT after 6 months (n = 119).

Predictor	HR	95% CI	P-value
**Univariate analysis**			
Age	0.994	0.964–1.028	0.749
Sex, female	0.602	0.263–1.203	0.185
Relative decline in body mass index, %	1.084	1.037–1.128	<0.001
Relative decline in FVC, % predicted	1.041	1.013–1.069	0.004
Relative decline in DL_CO_, % predicted*	1.013	0.991–1.034	0.248
Relative decline in ESM_CSA_, %	1.057	1.027–1.086	<0.001
**Multivariate analysis**			
Relative decline in body mass index, %	1.036	0.986–1.088	0.163
Relative decline in FVC, % predicted	1.021	0.992–1.050	0.155
Relative decline in ESM_CSA_, %	1.039	1.007–1.071	0.015

Abbreviations: HR, hazard ratio; CI, confidence interval; FVC, forced vital capacity; DLCO, diffuse capacity of the lung for carbon monoxide; ESMCSA, cross-sectional area of erector spinae muscles.

*We analysed using n = 114, because 5 cases were missing.

HRs and 95% CI for each parameter on uni- and multivariate Cox regression analyses in the baseline cohort (n = 144) are shown in Supplemental Table 3.

### Kaplan–Meier curves, log-rank tests and number at risk

**Figure 3:**
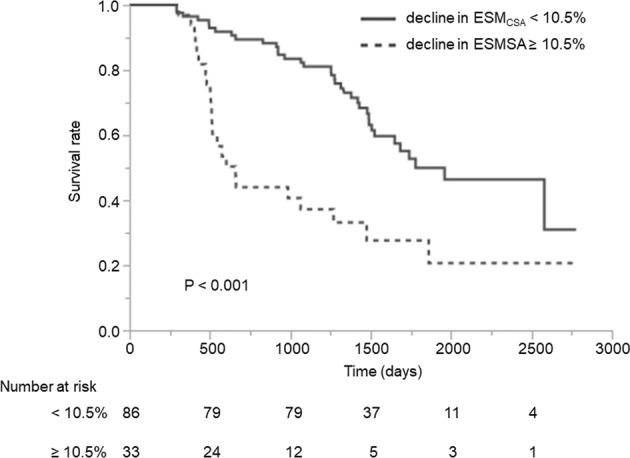
Kaplan–Meier curves and log-rank test. Kaplan–Meier survival curves stratified by the relative decline in ESM_CSA_ at 6 months (n = 119). The cutoff value was set at 10.5%. The patients with a relative decline in ESM_CSA_ more than 10.5% had significantly poorer survival (P < 0.001 by log-rank test).

Kaplan**–**Meier curves of the cohort available for 6 months of follow-up (n =119) are shown in Fig. 3. To determine an optimal cutoff value for predicting 3-year mortality, we constructed receiver operator characteristic (ROC) analysis. A cutoff value of 10.5% (AUC = 0.734, specificity: 0.752, sensitivity: 0.553) was identified. The median survival times were as follows: the relative decline in ESM_CSA_ ≥ 10.5%, 602 [480**–**1269] days; the relative decline in ESM_CSA_ < 10.5%, 1431 [1130**–**1743] days. The relative decline in ESM_CSA_ ≥ 10.5% had a significantly poorer prognosis (P < 0.001, log-rank test).

### Characteristics with IPF patients with or without ESM_CSA_ decline

The clinical characteristics of patients with IPF with (≥10.5%) or without (<10.5%) ESM_CSA_ decline are shown in Table 4. There were significant difference in baseline BMI (P = 0.049), baseline %FVC (P = 0.002), baseline forced expiratory volume in 1 second (FEV_1_)/FVC (P = 0.002), baseline lowest SpO_2_ during 6MWT (P = 0.004), ESM_CSA_ after 6 months (P < 0.001), survival time (P < 0.001), the relative decline in ESM_CSA_ (P < 0.001), the relative decline in %FVC (P = 0.003), the relative decline in %DL_CO_ (P = 0.004) and the relative decline in BMI (P < 0.001). During the 6 months, there were 7 unexpected hospitalisations (3 acute exacerbations, 2 infections and 2 instances of worsening IPF). Pirfenidone was prescribed to 28 patients during the 6 months whereas nintedanib was prescribed to none (Table 4). The number of cancer and chronic heart failure comorbidities, as well as, the Charlson comorbidity index of the study population, are also shown in Table 4.

**Table 4:** Characteristics with IPF patients with or without ESM_CSA_ decline.

Variable	With ESM_CSA_ decline (≥10.5%)	Without ESM_CSA_ decline (<10.5%)	P-value
Total, n	33	86	
Age, years	65.0 [60.0–71.0]	67.0 [62.0–70.8]	0.744
Sex, Female, n (%)	6 (18.2%)	15 (17.4%)	
Baseline body mass index, kg/m^2^	22.6 [21.5–24.1]	24.0 [21.8–26.0]	0.049
Baseline FVC, % predicted, %	73.0 [64.9–86.6]	87.2 [72.7–99.7]	0.002
Baseline FEV_1_/ FVC, %	89.7 [84.8–94.0]	85.5 [79.1–89.2]	0.002
Baseline DL_CO_, % predicted, %	60.0 [43.6–76.8]	60.8 [49.4–76.5]	0.690
Baseline distance walked during 6MWT, m	558 [482–627]	593 [536–649]	0.123
Baseline lowest SpO_2_ during 6MWT, %	80.0 [77.0–86.5]	87.0 [82.0–90.0]	0.004
Baseline ESM_CSA_, cm^2^	34.2 [28.2–37.5]	34.3 [27.3–40.7]	0.495
ESM_CSA_ after 6 months, cm^2^	27.4 [23.5–31.5]	33.6 [27.4–39.5]	<0.001
Survival time, day	602 [480–1269]	1430 [1130–1743]	<0.001
Death during observation period, n (%)	23 (69.7%)	36 (41.9%)	
Relative decline in ESM_CSA_, %	15.7 [13.9–21.8]	3.0 [−2.2–6.9]	<0.001
Relative decline in %FVC, %	4.0 [−1.5–12.8]	0.3 [−3.5–4.1]	0.003
Relative decline in %DLCO, %	6.4 [2.1–14.5]	1.4 [−4.2–7.8]	0.004
Relative decline in body mass index, %	3.2 [−1.0–10.5]	−0.1 [−3.3–2.2]	<0.001
Corticosteroid treatment, n (%)	5 (15.2%)	6 (7.0%)	
Pirfenidone treatment, n (%)	12 (35.3%)	16 (18.6%)	
Hospitalisation during 6 months, n (%)	4 (12.1%)	3 (3.5%)	
Acute exacerbations, n (%)	0 (0%)	3 (3.5%)	
Infections, n (%)	2 (6%)	0 (0%)	
Worsening of IPF	2 (6%)	0 (0%)	
Charlson comorbidity index	1.0 [1.0–1.0]	1.0 [1.0–1.0]	
Chronic heart failure, n (%)	2 (6%)	2 (6%)	
Cancers, n (%)	1 (3%)	2 (6%)	

Data are presented as median [interquartile range] or n (%). Abbreviations: ESMCSA, cross-sectional area of elector spine muscles; FVC, forced vital capacity: FEV1, forced expiratory volume in 1.0 second; DLCO, diffuse capacity of the lung for carbon monoxide; 6MWT; 6-minute walk test, SpO2; percutaneous oxygen saturation, IPF, idiopathic pulmonary fibrosis. *We analysed using n = 114, because 5 cases were missing.

### Decline in ESM_CSA_ and effects of treatment with corticosteroids

No patients had been treated with corticosteroids at baseline, but 11 patients were treated with corticosteroids 6 months later. We compared the relative decline in ESM_CSA_ between the patients treated with corticosteroids and those who were not. In the patients treated with corticosteroids, the relative decline in ESM_CSA_ was 9.6 [3.5–16.8] % after 6 months. In the patients not treated with corticosteroids, the relative decline in ESM_CSA_ was 6.0 [−0.2–10.6] % after 6 months. There was no statistical difference (P = 0.096) between the two groups (Wilcoxon rank-sum test).

## Discussion

Baseline ESM_CSA_ is a prognostic factor in patients with IPF^22,24^. Here, we demonstrated that the serial change in ESM_CSA_ is also a prognostic factor in patients with IPF. On multivariate analysis, the decline in ESM_CSA_ was a significant prognostic factor, while those of predicted %FVC and BMI were not.

It is widely known that the decline in FVC is a strong prognostic factor^15,16^. Measurements of serial changes in FVC are used as a gold standard in clinical trials^25–28^. Interestingly, in the present study, the decline in ESM_CSA_ (HR 1.039, 95% CI 1.007–1.071, P = 0.015) was a significant prognostic factor in the multivariate analyses, while the decline in %FVC was not. This result may indicate that the decline in ESM_CSA_ is a stronger prognostic factor than the decline in %FVC. The decline in ESM_CSA_ was correlated with the decline in %FVC. However, the decline in ESM_CSA_ (6.2 [0.5–11.8] %) was significantly greater than that in %FVC (1.4 [−3.1–5.0] %). It is possible that this result influences the data of multivariate Cox-proportion analysis. We did not evaluate the results of 6MWT because the missing data of 6 months later were not small. Further studies are needed to verify whether the decline in ESM_CSA_ is useful for predicting mortality as compared with serial changes in the lowest SpO_2_ and the distance walked during the 6MWT.

Cachexia and sarcopenia have been extensively studied in patients with lung cancer and COPD. Evan’s diagnostic criteria for cachexia include evaluation items such as muscle mass, fatigue and weight loss. In an attempt to include a wider evaluation of the burden of cachexia, diagnostic criteria based on an assessment of laboratory data and symptoms in addition to weight have been proposed^17^. The criteria included weight loss ≥5% in 12 months or low BMI (<20 kg/m^2^) with 3 of 5 of the following features: decrease muscle strength, fatigue, anorexia, low fat-free mass index, of abnormal biochemistry (increased inflammatory markers, anaemia and low serum albumin). We reason that cachexia would be associated with the survival in IPF.

Kinsey *et al*. reported that a smaller pectoralis muscle area, as measured on CT images obtained at the time of diagnosis of non-small cell lung cancer, was associated with poorer overall survival^29^. McDonald *et al*. reported that patients with a smaller pectoralis muscle area on CT scan tended to have a more severe expiratory air flow obstruction, lower quality of life scores and diminished exercise capacity, as compared with people with a lower BMI^30^. The measurement of the psoas muscle at the L3 or L4 level on CT images is frequently used for evaluating sarcopenia^31^. Canvasser *et al*. reported a strong correlation between the paraspinous muscle area at Th12 and the total psoas area at L4 (r = 0.72, P < 0.001), and both were associated with lower mortality rates after surgery^32^. Those authors suggest that measuring the area of the erector spinae muscles at the Th12 level might be useful for evaluating skeletal muscles in patients with lung disease who have not undergone an abdominal CT. However, no data on the pectoralis muscles exist in patients with IPF presently. In patients with IPF, chest CT scans are regularly taken in clinical practice, along with chest X-rays and pulmonary function tests. Based on these and our results, measuring erector spinae muscles (ESMs) by chest CT images would be useful in evaluating sarcopenia and cachexia in patients with IPF.

Loss of skeletal muscle mass is considered to be induced by systemic inflammation, inactivity, malnutrition and enhanced energy expenditure^17–20^. In the present study, the decline in ESM_CSA_ was weakly correlated with the decline in BMI (r = 0.394 [0.2170.552], P < 0.001), baseline lowest SpO_2_ during 6MWT (r = −0.290 [−0.458–−0.101], P = 0.002), baseline %FVC (r = −0.285 [−0.464–−0.089], P = 0.002) and the decline in %FVC (r = 0.202 [0.0220.358], P = 0.028). In a previous report, ESM_CSA_ was weakly correlated with percentage of predicted FEV_1_ (r = 0.31, P < 0.0004) in patients with COPD^21^. Impaired pulmonary function, exercise-induced hypoxemia and nutrition status might influence the decline in ESM_CSA_. However, further studies are needed to understand the mechanisms underlying muscle loss in IPF.

Only 11 patients were treated with corticosteroids in this study, and there was no significant difference in the decline in ESM_CSA_. However, we cannot rule out the possibility that muscle atrophy due to the side effects of corticosteroids affected the ESM_CSA_. Further studies are needed to explore this possibility. Moreover, the influence of appetite loss side effects of anti-fibrotic drugs on the ESM_CSA_ should be investigated in future studies.

Recently, several studies have reported the association between lung disease and ESM_CSA_. The ESM_CSA_ was significantly associated with health-related quality of life and prognostic physiological parameters in patients with *Mycobacterium avium* complex lung disease^33^. A smaller ESM_CSA_ was significantly associated with a lower level of activities of daily living at the end of treatment for pneumonia^34^.

The present study has the following limitations. First, in the study, the results were obtained by a retrospective analysis of all Japanese patients from a single centre. The sample size was small and there was no replication cohort. Further studies are required to confirm our observations in other external validation cohorts. Second, the diagnoses of IPF were according to the 2011 international guideline. The diagnosis of patients who initially presented before 2011 was confirmed by MDD according to the 2011 guidelines. In these patients, the diagnosis may be more accurate due to a consideration of the MDD time course. The limitation is that the MDD diagnosis before and after 2011 may not be the same. Third, 25 of 144 patients (17.4%) were not available for CT images to compare the changes at 6 months. Eleven patients died within 6 months, and CT images of the rest of the patients were not available for several reasons. Fourth, in the present study, approximately 16.7% (30 of 180 patients) of the CT of patients with IPF excluded the 12th thoracic spinous process level. This might be because the lungs of patients with IPF are smaller than those with COPD. This is another limitation of the present study. Fifth, we did not adjust for treatment in the survival analysis. The reason was that there was bias because a little evidence in anti-fibrotic drugs for IPF existed before 2014.

In conclusion, we have demonstrated that a smaller ESM_CSA_ is a prognostic predictor in patients with IPF. A reduced ESM_CSA_ after 6 months was an independent prognostic predictor in patients with IPF.

## Methods

This single centre retrospective study was performed in accordance with the amended Declaration of Helsinki. The ethical review board of Tosei General Hospital, which contributed cases to this study, provided approval for the study (approval number 745). The study was also approved by the ethical review board of the Nagoya City University Hospital (approval number 60-18-0210), where the CT analyses were performed. Given that the data were analysed anonymously, the ethical review boards did not ask for the patients’ approval or informed consent. The opt-out document can be found on the website of Tosei General Hospital.

### Patients

During the period of June 2008 to July 2013, the clinical records of 180 consecutive patients with IPF, referred to the Tosei General Hospital without corticosteroid or anti-fibrotic drug pre-treatments, were retrospectively reviewed. IPF was diagnosed by multidisciplinary discussion (MDD) according to 2011 international guidelines^1^. The diagnosis of patients who initially presented before 2011 was confirmed by MDD according to the 2011 guidelines before May 2015.

The following patients were excluded: one patient, who experienced acute exacerbations at the initial visit; patients who had other comorbidities, such as lung cancer (n = 1), infectious diseases or congested cardiac failure at the initial presentation; thirty patients, whose chest CT lacked the level of the spinous process of the 12th thoracic vertebrae and four patients, who underwent lung transplantation because due to the small number of patients and the consideration of death and lung transplantation as different outcomes.

Ultimately a total of 144 patients were enrolled. After approximately 6 months, Chest CT images were available for 119 patients. The interval between performing the CT at baseline and the second time was 190 [169–217] days.

Besides, the duration between the day of performing the chest CT on the first visit to the last visit or death was recorded. Other cases were contacted to confirm their life-or-death status by telephone.

### Computed tomography

All patients underwent CT scan using a commercially available CT scanner (Aquilion, Toshiba Medical Systems, Tokyo, Japan) with a high-frequency algorithm. HRCT images were obtained without intravenous contrast and with the patient in the supine position at full inspiration. HRCT images with 0.5-mm-thick slices at 0.5-mm intervals were used for the analysis.

### Derivation of ESM_CSA_ by imaging analysis software

**Figure 4:**
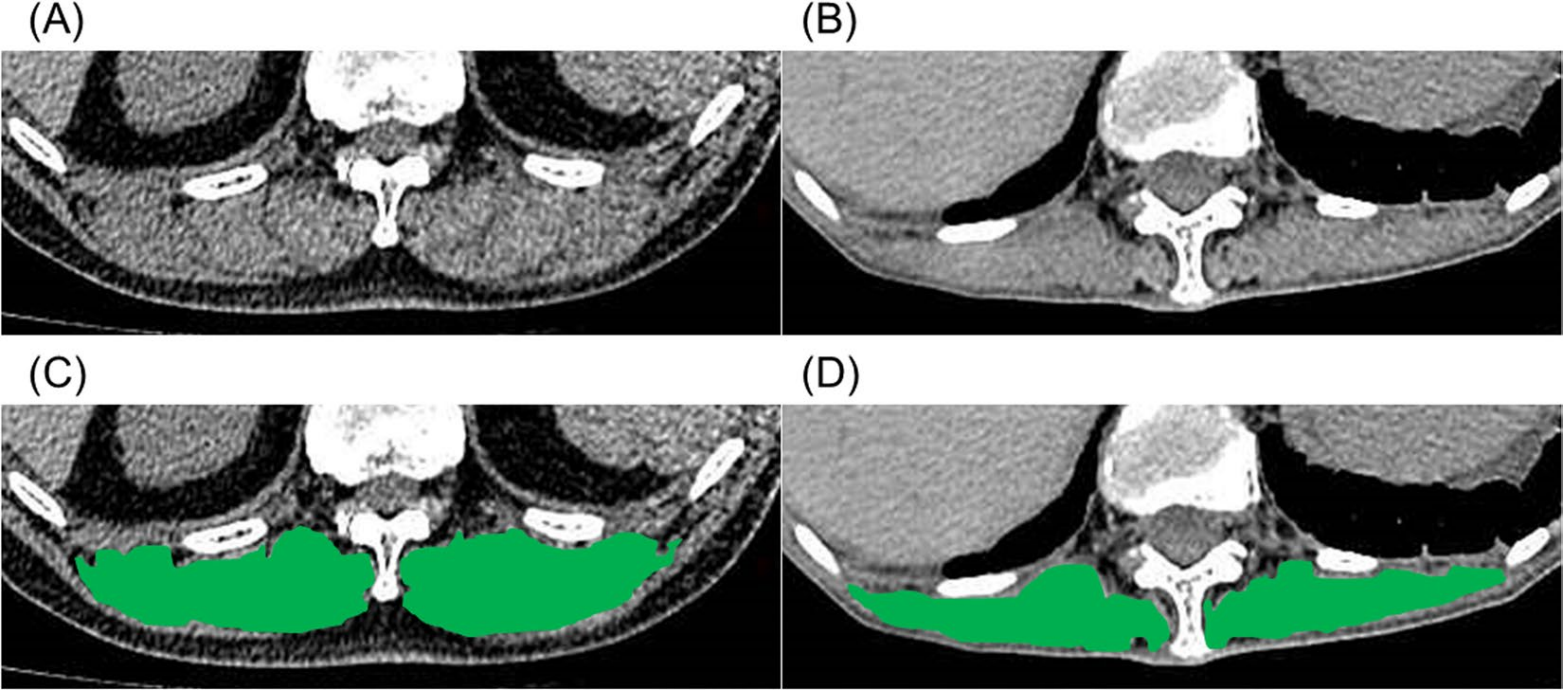
The cross-sectional area of the erector spinae muscles. Representative computed tomographic images used to measure the cross-sectional area of the erector spinae muscles (**A**,**B**). The cross-sectional areas of the erector spinae muscles are in green (**C**,**D**). The sums of the areas of the erector spinae muscles were 55.8 cm^2^ (**A**,**C**), and 14.9 cm^2^ (**B**,**D**).

SYNAPSE VINCENT (Fujifilm Medical Systems, Tokyo, Japan) CT imaging analysis software was used for the derivation of ESM_CSA_. ESM_CSA_ was calculated manually according to a previously published method^21^. Briefly, ESM_CSA_ was measured on a single-slice axial CT image at the level of the spinous process of the 12th thoracic vertebra. For the quantitative analysis of the ESMs, chest HRCT images were reconstructed using the mediastinal window settings (window level, 40 HU; window width, 300 HU). The left and right ESMs were identified and manually shaded and the ESM area was reported as the sum of the right and left ESMs. All CT analyses were independently performed by trained individuals (ANa and HO) who were blinded to the patients’ clinical information. The average values of ESM_CSA_ of ANa and HO were used in this study. Figure 4 shows the images of the ESM_CSA_ in two patients with IPF. The correlation of the values of ESM_CSA_ between trained individuals and the agreement of two individuals were analysed using the Spearman’s correlation test and the Bland-Altman analysis, respectively.

### Pulmonary function tests and six-minute walk test

All patients underwent pulmonary function tests by spirometry (CHESTAC-55 V; Chest, Tokyo, Japan), according to the American Thoracic Society and European Respiratory Society (ATS/ERS) criteria^35^. The DL_CO_ was also measured (CHESTAC-55 V). The values of FVC, FEV_1_ and DL_CO_ were measured according to ATS/ERS recommendations^36^. We calculated %FEV_1_, %FVC and %DL_CO_ based on the patients’ height, age and sex per the Japanese guidelines^37^. We then conducted 6MWTs without supplemental oxygen in accordance with ATS guidelines^38^.

### The comorbidities and the Charlson comorbidity index

The Charlson comorbidity index was calculated according to a previously reported method^39^.

### Statistical analyses

Continuous variables were presented as medians and interquartile ranges. Categorical variables were presented as numbers and percentages. The differences between patients who underwent HRCT after 6 months and those who did not were analysed using the Student’s t-test or the Wilcoxon rank-sum test. Spearman’s rank correlation coefficients were used to test for correlations between ESM_CSA_ and clinical parameters and those between the relative decline in ESM_CSA_ and clinical parameters. Univariate and multivariate Cox regression analyses were performed to evaluate the relationship between each variable and mortality. We included factors with P-values < 0.05 in the univariate analysis for the multivariate analyses. Survival times were estimated using the Kaplan–Meier method and compared with the log-rank test. To determine the optimal cutoff value for predicting 3-year mortality, we constructed ROC curves. P-values less than 0.05 were considered significant. The statistical analyses were conducted using JMP statistical software (version 14; SAS Institution Japan Ltd, Japan). Since we could not analyse the r (95%CI) with JMP statistical software (version 14), we analysed r (95%CI) using SPSS (version 26; IBM, Japan).

## Supplementary information

Supplementary information
