# Supplementary material for: Early decrease in erector spinae muscle area and future risk of mortality in idiopathic pulmonary fibrosis
Source: Sci Rep. 2020 Feb 11;10:2312. doi: 10.1038/s41598-020-59100-5 (PMC7012911; doi:10.1038/s41598-020-59100-5)
Supplement: Supplementary file 1 — Supplementary information [file 41598_2020_59100_MOESM1_ESM.docx]

**Early decrease in erector spinae muscle area and future risk of mortality in idiopathic pulmonary fibrosis**

***：Online Supplement***

**Authors:** Akiko Nakano M.D.^a^, Hirotsugu Ohkubo M.D., Ph.D.^a^, Hiroyuki Taniguchi M.D., Ph.D.^b^, Yasuhiro Kondoh M.D., Ph.D.^b^, Toshiaki Matsuda M.D., Ph.D.^b^, Mitsuaki Yagi M.D., Ph.D.^c^, Taiki Furukawa M.D.^d^, Yoshihiro Kanemitsu M.D., Ph.D.^a^ and Akio Niimi M.D., Ph.D.^a^

**Institutional affiliation:**

a) Department of Respiratory Medicine, Allergy and Clinical Immunology, Nagoya City University Graduate School of Medical Sciences, Nagoya, Japan.

b) Department of Respiratory Medicine and Allergy, Tosei General Hospital, Japan.

c) Department of Respiratory Medicine, National Hospital Organization Higashinagoya National Hospital, Nagoya, Japan.

d) Department of Respiratory Medicine, Nagoya University Graduate School of Medicine, Nagoya, Japan.

**Corresponding author:** Hirotsugu Ohkubo, Department of Respiratory Medicine, Allergy and Clinical Immunology, Nagoya City University Graduate School of Medical Sciences, 1 Kawasumi, Mizuho-cho, Mizuho-ku, Nagoya, Aichi 467-8601, Japan, **Fax:** +81-52-852-0849, **Phone:** +81-52-853-8216, **E-mail:** hohkubo@med.nagoya-cu.ac.jp

**Supplemental Table 1. Patient characteristics and ESM_CSA_**

| Variable | Baseline  (n = 144) | Taken HRCT  after 6 months  (n = 119) | Not taken HRCT after 6 months  (n = 25) | P value  * |
| --- | --- | --- | --- | --- |
| Age, years | 68.0 [62.0−72.0] | 67.0 [61.0−71.0] | 72.0 [69.0−75.0] | 0.001 |
| Sex, Female, n (%) | 26 (18.1%) | 21 (17.6%) | 5 (20.0%) |  |
| Never smoker, n (%) | 33 (22.9%) | 25 (21.0%) | 8 (32.0%) |  |
| Ex-smoker, n (%) | 95 (66.0%) | 81 (68.1%) | 14 (56.0%) |  |
| Current smoker, n (%) | 16 (11.1%) | 13 (10.9%) | 3 (12.0%) |  |
| Smoking history, pack-years | 34.0 [3.4−54.0] | 36.0 [5.0−55.5] | 30.0 [0−50.0] | 0.116 |
| Body mass index, kg/m^2^ | 23.2 [21.3−25.1] | 23.4 [21.7−25.2] | 22.0 [19.5−25.0] | 0.039 |
| Biopsy-proven IPF, n (%) | 69 (47.9%) | 62 (52.1%) | 7 (28.0%) |  |
| FVC, % predicted | 80.4 [68.5−93.8] | 84.2 [70.4−96.5] | 67.0 [56.6−76.6] | < 0.001 |
| FEV_1_/ FVC, % | 85.8 [81.6−90.8] | 85.8 [81.3−90.6] | 87.4 [83.3−92.4] | 0.166 |
| DL_CO_, % predicted** | 59.8 [47.2−72.8] | 60.7 [48.8−76.7] | 52.3 [40.0−59.6] | 0.008 |
| Distance walked during 6MWT, m | 570 [511−637] | 589 [524−645] | 503 [395−561] | < 0.001 |
| Lowest SpO_2_ during 6MWT, % | 85.0 [78.0−89.0] | 85.0 [80.0−89.0] | 80.0 [75.0−87.0] | 0.046 |
| ESM_CSA_, cm^2^ | 33.3 [25.9−39.1] | 31.6 [25.0−37.0] | 26.8 [22.6−34.3] | 0.009 |

Data are presented as median [interquartile range] or n (%). Abbreviations: ESM_CSA_, cross-sectional area of elector spine muscles; FVC, forced vital capacity; FEV_1_, forced expiratory volume in 1.0 second; DL_CO_, diffuse capacity of the lung for carbon monoxide; 6MWT, 6-minute walk test; SpO_2_, percutaneous oxygen saturation. * P values are reported for the differences between patients who underwent HRCT after 6 months or not, using Student’s-t test or the Wilcoxon rank sum test.

**We analysed using n = 140, because 4 cases were missing.

**Supplemental Table 2. Correlations between baseline ESM_CSA_ in 144 patients and other clinical parameters.**

| Variables | *r* | 95%CI | P-value |
| --- | --- | --- | --- |
| Age, years | −0.331 | −0.474 − 0.162 | <0.001 |
| Body mass index, kg/m^2^ | 0.398 | 0.240 – 0.548 | <0.001 |
| FVC, % predicted | 0.342 | 0.084 – 0.437 | <0.001 |
| FEV_1_/FVC, % | −0.299 | −0.448 − −0.134 | <0.001 |
| DL_CO,_ % predicted* | 0.032 | −0.195 – 0.167 | 0.707 |
| Distance walked during 6MWT, m | 0.306 | 0.101 −0.413 | < 0.001 |
| Lowest SpO_2_ during 6MWT, % | −0.009 | −0.218 – 0.141 | 0.912 |

Abbreviations: ESM_CSA_, cross-sectional area of elector spine muscles; CI, confidence interval; FVC, forced vital capacity; FEV_1_, forced expiratory volume in 1.0 second; DL_CO_, diffuse capacity of the lung for carbon monoxide; 6MWT, 6-minute walk test; SpO_2_, percutaneous oxygen saturation.

*We analysed using n = 140, because 4 cases were missing.

**Supplemental Table 3. Prediction of mortality by uni- and multivariate Cox-proportion analyses in the baseline cohort (n=144)**

| Predictor | HR | 95% CI | P-value |
| --- | --- | --- | --- |
| Univariate analysis |  |  |  |
| Age, years | 1.011 | 0.983–1.040 | 0.461 |
| Sex, female | 0.640 | 0.319–1.163 | 0.151 |
| Body mass index, kg/m^2^ | 0.946 | 0.875–1.024 | 0.170 |
| FVC, % predicted | 0.952 | 0.938–0.967 | < 0.001 |
| DL_CO_, % predicted* | 0.977 | 0.964–0.989 | < 0.001 |
| Distance walked during 6MWT, m | 0.997 | 0.994–0.998 | < 0.001 |
| Lowest SpO_2_ during 6MWT, % | 0.924 | 0.897–0.952 | < 0.001 |
| ESM_CSA_, cm^2^ | 0.973 | 0.947–0.998 | 0.033 |
|  |  |  |  |
| Multivariate analysis |  |  |  |
| FVC, % predicted | 0.965 | 0.947–0.983 | < 0.001 |
| DL_CO_, % predicted* | 1.000 | 0.983–1.018 | 0.993 |
| Distance walked during 6MWT, m | 0.999 | 0.996–1.002 | 0.469 |
| Lowest SpO_2_ during 6MWT, % | 0.960 | 0.924–0.998 | 0.038 |
| ESM_CSA_, cm^2^ | 0.991 | 0.961–1.022 | 0.579 |

Abbreviations: HR, hazard ratio; CI, confidence interval; FVC, forced vital capacity; DL_CO_, diffuse capacity of the lung for carbon monoxide; 6MWT, 6-minute walk test; SpO_2_, percutaneous oxygen saturation; ESM_CSA_, cross-sectional area of elector spine muscle.

* We analysed using n = 140, because 4 cases were miss
